# Characterization of electrocorticographic, electromyographic and electrocardiographic recordings after the use of caffeine in Wistar rats

**DOI:** 10.31744/einstein_journal/2021AO6417

**Published:** 2021-11-03

**Authors:** Diego Arthur Castro Cabral, Fernanda Myllena Sousa Campos, Maria Clara Pinheiro da Silva, João Paulo do Vale Medeiros, Paula dos Santos Batista, Giovanna Coutinho Jardim, Jéssica Lígia Picanço Machado, Leonardo Giovanni Castro Cabral, Vanessa Joia de Mello, Moises Hamoy

**Affiliations:** 1 Universidade Federal do Pará BelémPA Brazil Universidade Federal do Pará, Belém, PA, Brazil.

**Keywords:** Caffeine, Electrocorticography, Electromyography, Electrocardiography, Central nervous system, Rats, Wistar

## Abstract

**Objective:**

To describe electrocorticographic, electromyographic and electrocardiographic profiles to report the electrophysiological effects of caffeine in Wistar rats.

**Methods:**

Male adult Wistar rats weighing 230g to 250g were used. Rats were allocated to one of two groups, as follows: Group 1, Control, intraperitoneal injection of 0.9% saline solution (n=27); and Group 2, treated with intraperitoneal injection of caffeine (50mg/kg; n=27). The rats were submitted to electrocorticographic, electromyographic and electrocardiographic assessment.

**Results:**

Brain oscillations (delta, theta, alpha, beta and gamma) in the frequency range up to 40Hz varied after caffeine administration to rats. Powers in delta and theta oscillations ranges were preponderant. The contractile force of the skeletal striated and cardiac muscles increased. Electrocardiogram analysis revealed shorter RR, QRS and QT intervals under the effect of caffeine.

**Conclusion:**

In the central nervous system, there was an increase in the delta, theta and alpha amplitude spectrum, which are related to memory encoding and enhanced learning. With regard to skeletal muscle, increased contraction of the gastrocnemius muscle was demonstrated, a clear indication of how caffeine can be used to enhance performance of some physical activities. Electrocardiographic changes observed after caffeine administration are primarily related to increased heart rate and energy consumption.

## INTRODUCTION

Caffeine is a central nervous system (CNS) stimulant of the methylxanthine class, and the most widely used psychoactive drug worldwide. Motivations behind caffeine use are increased concentration, cognition and physical performance.^( 1 )^ Caffeine can be used to treat idiopathic apnea of prematurity^( 2 )^ and acute respiratory depression,^( 3 )^ and for pain management.^( 4 )^ Epidemiological data suggest that habitual coffee consumption is protective against Parkinson's and Alzheimer's diseases and promotes weight loss.^( 4 , 5 )^

Caffeine acts primarily as a non-selective adenosine receptor antagonist. Caffeine increases motor activity and has arousal and reinforcing effects.^( 6 )^ Paraxanthine is the main metabolite of caffeine in humans and is associated with a significant release of dopamine in areas of the striatum.^( 7 )^

The stimulating effect of caffeine has been widely described in literature, primarily in behavioral and biochemical studies.^( 1 , 6 , 8 )^ However, quantitative studies describing the impact of electrophysiological changes on various systems following caffeine administration are scarce.

## OBJECTIVE

To describe the electrophysiological changes induced by caffeine in male Wistar rats, based on electrocorticographic, electromyographic and electrocardiographic recordings.

## METHODS

### Animals

The animals were obtained from the Central Animal Facility of *Universidade Federal do Pará* (UFPA), and individually housed in the Experimentation Vivarium of the Laboratory of Pharmacology and Toxicology of Natural Products, from April 2019 to November 2020. Animals had access to water and food *ad libitum* , and were kept in a temperature-controlled environment (25°C to 28°C), under a 12/12 hour light-dark cycle. Experimental procedures were conducted in compliance with the principles of laboratory animal care and approved by the Ethics Committee on Experiments in Animals (CEUA No. 2675110219).

A total of 54 adult male Wistar rats, weighing 230g to 250g, were used. Rats were allocated to one of two groups, as follows: Group 1 (n=27), Control, treated with equivalent volume of 0.9% saline solution in an intraperitoneal (IP) injection; Group 2 (n=27), Caffeine with Treated (50mg/kg IP), *as per* Marriott, 1968.^( 9 )^ Rats were submitted to electrocorticographic (ECoG), electromyographic (EMG) and electrocardiographic (ECG) assessment. Surgically implanted electrodes were used to record ECoG, ECG and EMG signals. Data were collected in separate groups, within 5^th^ days of surgery. Measurements were made on the same day, using a block design, as follows: ECoG data were collected first, then ECG, and finally EMG data. Rats were not anesthetized prior to recordings. Rats were placed in acrylic boxes measuring 60x50x20cm (length, width and height, respectively).

### Chemicals

The following chemicals were used: ketamine hydrochloride (Laboratório Köing, Santana de Parnaíba, SP, Brazil), xylazine hydrochloride (Laboratório Vallée, Montes Claros, MG, Brazil), lidocaine (Laboratório Hipolabor, Sabará, MG, Brazil) and caffeine powder (Sigma), in the form of crystals diluted and 0.9% saline solution.

### Electrode implantation surgery

Surgical procedures were performed under general anesthesia obtained with 5mg/kg of xylazine and 50mg/kg of ketamine. The site of electrode implantation was anesthetized with lidocaine. Anesthetized rats were placed in a stereotactic device. After proper positioning, the implantation site was clipped, and an incision made through the skin, subcutaneous tissue and muscle planes to access the skull. Two small craniotomies were then created, −0.96 and 1mm lateral to the midline bony landmark (bregma), to access each cerebral hemisphere containing the motor cortex, where electrodes were implanted into the brain surface.^( 10 )^ A screw was inserted at the craniotomy site for electrode attachment using dental acrylic resin.

### Electrocorticogram

Animal preparation and electrode implantation procedures for ECoG acquisition were based on previous publications.^( 11 )^ Electrodes were placed −0.96mm and 1mm lateral to the stereotaxic coordinate taken from bregma, in each hemisphere comprising the motor cortex.^( 10 )^ Recording and reference electrodes were located on the right and left hemisphere, respectively. On postoperative 5^th^ day electrodes were connected to a data acquisition system consisting of a high impedance amplifier (P511, Grass Technologies), monitored using an oscilloscope (Protek, 6510).^( 12 )^ Caffeine was injected IP, 15 minutes prior to ECoG recordings. Electrocorticographic data were continuously digitized at a rate of 1kHz, using a computer equipped with data acquisition board (National Instruments, Austin, TX, United States). Data were stored on a hard disk and processed using dedicated software (LabVIEWexpress). The entire experiment was carried out in a Faraday cage.

### Electromyogram

Following ECoG data acquisition, conjugate electrodes were implanted 0.5cm above the insertion of the gastrocnemius muscle, as described in previous studies.^( 13 )^ Intraperitoneal caffeine administration was performed 15 minutes prior to electromyographic recordings. Animals were kept in an acrylic box throughout the 10-minute recording procedure. Recordings were made with electrodes connected to a Grass P511 amplifier, monitored using an oscilloscope.^( 13 )^

### Electrocardiogram

Electrode insertion was guided by the vector plotted from lead D2. The reference electrode was placed in the region of the fourth intercostal muscle near the right axilla and the recording electrode in the 11^th^ intercostal space, 1.5cm to the left of the mid-sagittal line.^( 14 )^ Following intraperitoneal administration of 50mg/kg of caffeine and a 15-minute latency period, ECG data were recorded for 10 minutes per animal. The following variables were analyzed: amplitude (mV), heart rate (bpm), RR interval, PQ interval, QT interval and QRS duration.

### Electrophysiological data analysis

Amplitude graphs show potential differences between reference and recording electrodes at a sampling rate of one thousand samples per second. Spectrograms were calculated using a Hamming window with 256 points (256/1000 seconds); each frame was generated with an overlap of 128 points per window. For each frame, the spectral power distribution (SPD) was calculated using the Welch average periodogram method. A frequency histogram was generated from the first signal SPD calculation using a Hamming window with 256 points and no SPD overlap; this resulted in a histogram constructed with 1Hz boxes. Signals recorded up to 50Hz were analyzed. Frequency ranges were analyzed as follows: delta (1Hz to 4Hz), theta (4Hz to 8Hz), alpha (8Hz to 12Hz), beta (12Hz to 28Hz) and gamma (28Hz to 40Hz).^( 15 )^

### Statistical analysis

Data normality and homogeneity of variance were verified using the Kolmogorov-Smirnov and Levene's test respectively. Data were expressed as means and standard deviations; F and p values were provided when applicable. The level of significance was set at p<0.05. Groups were compared using two-way analysis of variance (ANOVA) followed by the Tukey's test for multiple comparisons. The Student's *t* -test was used to compare ECoG, EMG and ECG data (power recorded in different frequency bands) between the Control and the Caffeine with Treated Group. Analysis of variance was used exclusively to analyze the preponderance of frequency spectra within groups. Statistical analyses for outlier detection and removal were conducted using GraphPad Prism, version 8 (Graph-Pad Software Inc., San Diego, CA, United States).^( 12 )^

## RESULTS

### Caffeine altered the power spectrum of brain waves

**Figure 1:**
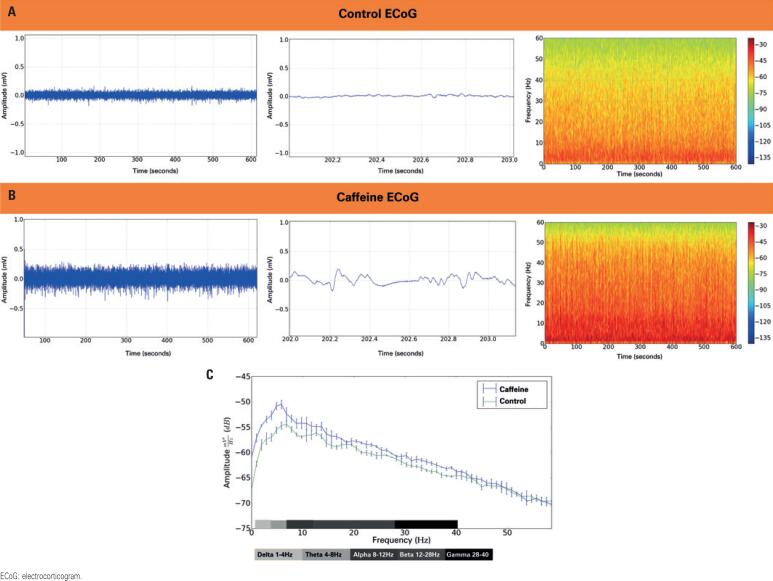
Recording of electrocorticograms in the Control Group and the Caffeine-Treated Group. A) Electrocorticogram of the Control Group, with expansion of the tracing that reveals power intensity at frequencies below 10Hz; B) Electrocorticographic recording obtained after intraperitoneal administration of 50mg/kg of caffeine where there is a predominance of frequencies above 10Hz; C) Spectral energy distribution after intraperitoneal administration of caffeine (50mg/kg) or saline solution (control) and their respective cerebral oscillations. Data were analyzed by comparing means, using the *t* test followed by the Mann-Whitney test, with a significance level of p<0.001 (n=9)

Significant changes in ECoG tracings were observed following caffeine administration relative to baseline ( Table 1 ). Control Group recordings figure 1A revealed greater amplitude and power intensity in the spectrogram at frequencies lower than 10Hz. Caffeine with Treated Group ECoG tracings figure 1B shows greater power distribution above 10Hz.

**Table 1:** Numerical values obtained from electrocorticographic records performed during the experiments

Group	Delta (1Hz-4Hz)	Theta (4Hz-8Hz)	Alpha (8Hz-12Hz)	Beta (12Hz-28Hz)	Gamma (28Hz-30Hz)
Control, mV^2^/Hzx10^-3^	0.003883±0.0009877	0.01440±0.002569	0.01176±0.001643	0.007670±0.001497	0.003218±0.002155
Caffeine with Treated, mV^2^/Hzx10^-3^	0.01227±0.001844 *	0.03031±0.006081 *	0.02189±0.003489 †	0.01979±0.003748 †	0.01173±0.001083 †

* Means p<0.001 and

† <0.05 relative to the Control Group.

All delta, theta, alpha, beta and gamma wave values obtained in the Control and the Caffeine with Treated Group are shown.

Brain wave distribution was shown in tracings recorded at baseline and under the effect of caffeine ( Figure 1C ). An increase in the power of low frequency waves was seen, especially those up to 40Hz (p<0.001). Overall, the greatest amplitude of the signal power spectrum fell in the 1Hz to 8Hz range ( *i.e* ., delta and theta wave ranges (p<0.001).

**Figure 2:**
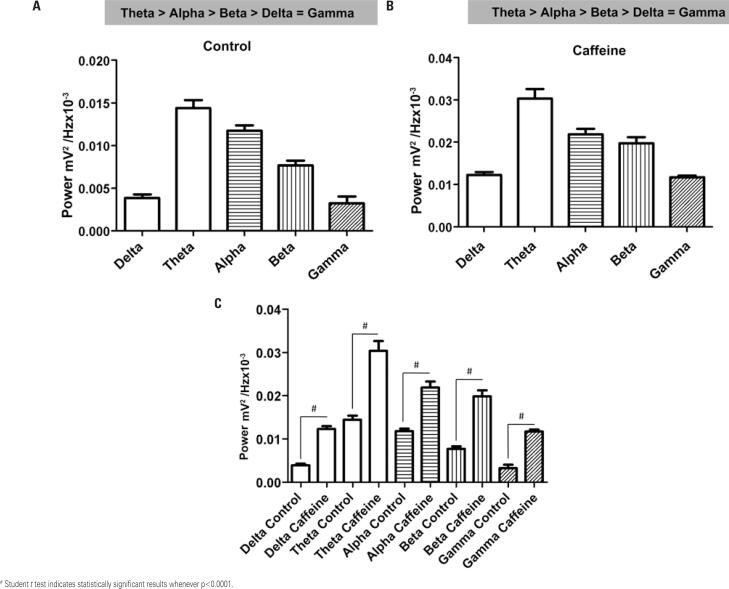
Average power range of delta, theta, alpha, beta and gamma brain oscillations in the Control and Caffeine-Treated Groups (50mg/kg). A) Powers of the predominant brain waves in the Control Group; B) Predominant potencies in brain oscillations in animals that received intraperitoneal caffeine; C) Comparison of the oscillations detected in each group (n=9)

The power spectrum of the Control Group revealed the following relationship: theta > alpha > beta > delta = gamma (p<0.05) ( Figure 2A ). In the Caffeine with Treated Group, the prevailing oscillation profile was theta > alpha = beta > delta = gamma (p<0.05) ( Figure 2B ).

Mean delta oscillation differed significantly prior to and after caffeine administration (0.003883±0.0009877mV^2^/Hzx10^-3^ and 0.01227±0.001844mV^2^/Hzx10^-3^, respectively; p<0.0001), with higher values detected in Caffeine with Treated Group, Mean oscillations in the theta (0.01440±0.002569mV^2^/Hzx10^-3^) and alpha (0.01176±0.001643mV^2^/ Hzx10^-3^) ranges in the Control Group differed significantly (p<0.001) from those recorded in the Caffeine with Treated Group (theta, 0.03031±0.006081mV^2^/Hzx10^-3^; alpha, 0.02189±0.003489mV^2^/Hzx10^-3^). Mean oscillations in the beta range differed significantly between the Control and the Caffeine with Treated Group (0.007670±0.001497mV^2^/Hzx10^-3^ and 0.01979±0.003748mV^2^/Hzx10^-3^ respectively; p<0.001). Mean oscillation in the gamma range corresponded to 0.003218±0.002155mV^2^/Hzx10^-3^ and 0.01173±0.001083mV^2^/Hzx10^-3^ in the Control Group and the Caffeine with Treated Group, respectively (p<0.001).

### Caffeine increased the amplitude of striated muscle contraction

**Figure 3:**
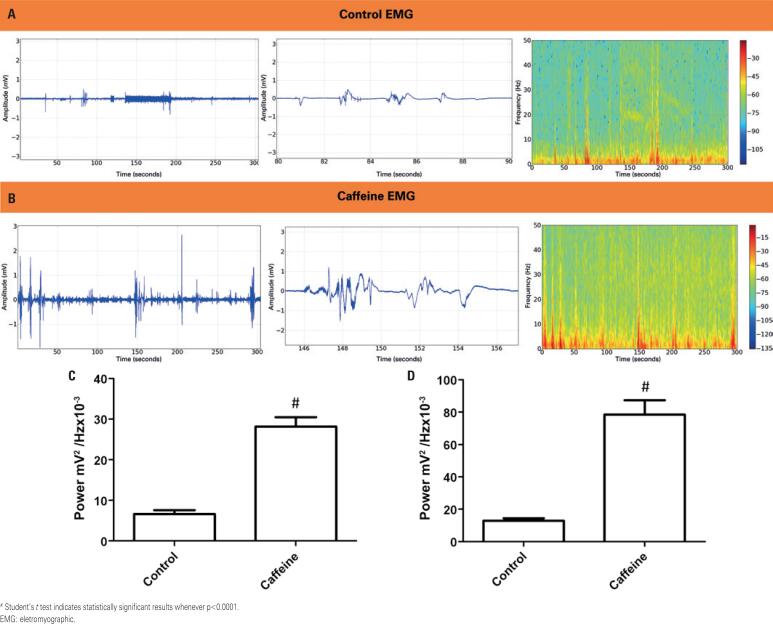
Electroneuromyographic recording of gastrocnemius muscle contraction. The registration time was 300 seconds. A) Registration of animals in the Control Group; B) Registration after caffeine administration; C) The power observed in the complete recording at frequencies up to 50Hz demonstrates the power of muscle contractions measured in control and caffeine-treated animals; D) Power of the strongest contractions recorded in the Control and Treated Caffeine Groups, at a fixed time of 5 seconds of contraction (n=9)

Electromyographic recordings in figure 3A and table 2 show muscle contraction patterns at low amplitude (up to 1mV), with an energy distribution spectrogram up to 50Hz. Within 15 minutes of intraperiotoneal administration of 50mg/kg of caffeine, contractions became more frequent, with amplitude larger than 2mV and higher energy concentration figure 3B .

**Table 2:** Numerical representation of the values obtained during the electromyographic study

Group	Control (mV^2^/Hzx10^-3^)	Caffeine (mV^2^/Hzx10^-3^)
Full EMG record	6.676±2.702	28.22±6.736
Strongest contraction recorded (EMG)	12.94±4.470 *	78.56±26.46 †

* Means p<0.05 and;

† <0.01 relative to the Control Group.

EMG: electromyographic.

All values recorded in the Caffeine with Treated and Control Groups are shown. Values recorded throughout electromyographic study and values recorded during maximal gastrocnemius muscle contraction are also reported.

Amplitude differences between recordings up to 50Hz shown in figure 3 indicate significant differences between the Control and the Caffeine with Treated Group (mean power 6.676±2.702mV^2^/Hzx10^-3^ and 28.22±6.736mV^2^/Hzx10^-3^ respectively; p<0.001) ( Figure 3C ). Analysis of the strongest contractions recorded in the Control and the Caffeine with Treated Group also revealed significant differences (12.94±4.470mV^2^/Hzx10^-3^ and 78.56±26.46mV^2^/Hzx10^-3^; p<0.001) ( Figure 3D ).

### Caffeine affected electrocardiographic parameters

**Figure 4:**
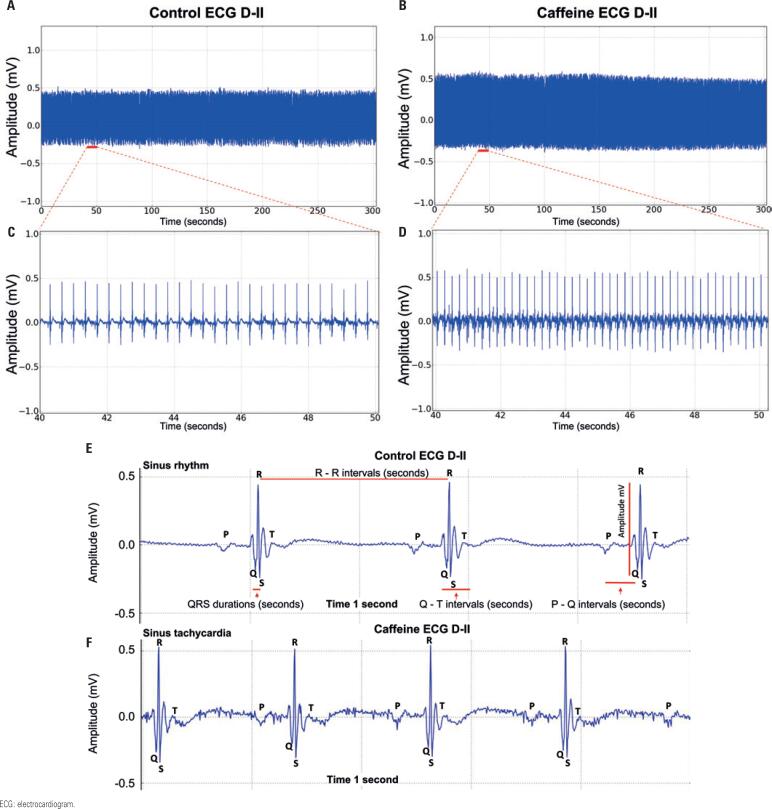
Animals' electrocardiogram and enlargement of the electrocardiographic tracing. A) Control group tracing in lead D-II; B) Electrocardiogram of the Caffeine-treated Group in lead D-II; C) Expansion of the interval from 40 to 50 seconds for the Control Group; D) The interval between 40 and 50 seconds of the electrocardiogram of animals treated with caffeine is extended; E) Electrocardiogram tracing of rats in sinus rhythm in lead D-II, showing the analyzed intervals, 1 second of increase; F) Electrocardiographic tracing of the Group Treated with Caffeine, demonstrating the characteristics of cardiac deflagration and shortening of the R-R interval, 1 second of increase

Electrocardiographic changes observed after caffeine administration were primarily related heart rate increase, as shown in figures 4A, B, C and D . Caffeine administration led to a significant increase in heart rate (249.2±21.26bpm and 303.7±7.194bpm, Control and Caffeine with Treated Group, respectively; p=0.0004) ( Figure 4E and Table 3 ).

**Table 3:** Numerical presentation of the values obtained during the execution of the electrocardiographic study

Parameter	Heart rate (bpm)	Amplitude (mV)	RR Intervals (seconds)	QRS duration (seconds)	QT intervals (seconds)	PQ intervals (seconds)
Control	249.2±21.26	0.4746±0.04072	0.3453±0.01276	0.01011±0.001364	0.0570±0.004062	0.07056±0.003779
Caffeine with Treated	303.7±7.194 *	0.5250±0.01949 †	0.2400±0.03651 *	0.0082±0.0007953 †	0.03153±0.004996 *	0.08306±0.007435 *

* p<0.001 relative to the Control Group and

† p<0.01 relative to the Control Group.

Parameters extracted from electrocardiographic records in the Control and Caffeine with Treated Group (Student's t test and Mann-Whitney test).

The mean amplitude also increased significantly after caffeine administration (0.4746±0.04072mV and 0.5250±0.01949mV, Control and Caffeine with Treated Group respectively; p=0.0061). The increase in heart rate revealed shortening of the RR interval in the ECG ( Figures 4E and F ); means differed significantly between the Control and the Caffeine with Treated Groups (0.3453±0.01276 seconds and 0.2400±0.03651 seconds, respectively; p=0.0004). The mean duration of the QRS complex was also characterized by a decrease in execution time and differed significantly between the Control and the Caffeine with Treated Groups (0.01011±0.001364 seconds and 0.0082±0.0007953 seconds, respectively; p=0.0062). The cardiac cycle represented by the QT interval, which involves the period of ventricular depolarization and repolarization, also differed significantly (0.0570±0.004062 seconds and 0.03153±0.004996 seconds, Control and Caffeine with Treated Group respectively; p=0.0004). As to the PQ interval, mean was 0.07056±0.003779 seconds in the Control Group and 0.08306±0.007435 seconds the Caffeine with Treated Group ( Table 3 ).

## DISCUSSION

In this study, ECoG, EMG and ECG recordings were used to describe electrophysiological changes in rats following caffeine administration. The average power was 50% higher in the amplitude of the delta brain oscillations in the Group Treated with Caffeine relative to the Control Group. Delta waves are thought to help encode memories and enhance learning.^( 16 )^ Positive acute effects on attention have also been demonstrated in most studies investigating the effects of caffeine on cognition.^( 17 )^ This finding may also be related to the delta stage, since delta activity “modulates” mental performance via inhibition of stimuli unrelated to the task at hand, thereby increasing the individual level of attention during execution of tasks that demand careful internal brain processing.^( 18 )^

The theta rhythm is implicated in several activities, such as establishment of word pattern for speech recognition and microsaccadic eye movement synchronization, which are often observed in the context of attentive and exploratory behavior and in implicit learning, a largely unconscious non-hippocampus-dependent learning category.^( 19 – 21 )^

Alpha waves are related to cognitive processing and self-regulation and are increased in situations associated with attention gains.^( 22 , 23 )^ Beta oscillations are a strong predictor of perceptual and motor performance.^( 24 )^ These oscillations are associated with states of alertness, focus and active thinking.^( 25 )^ In the upper cortex, gamma waves are enhanced during working memory and learning. Such oscillation plays a role in neural communication, reflecting the transfer of information from the external world to the brain.^( 26 )^

Increased amplitude of all brain waves in this study ( Figure 1C ) suggests caffeine or any of its metabolites may have direct or indirect impacts on pathways involved in the generation of such rhythms, which may enhance cognitive functions associated with brain oscillations. This finding supports the fact that caffeine acts as a CNS stimulant.^( 6 )^

In this study, intraperitoneal administration of caffeine increased the frequency of gastrocnemius muscle contraction, with higher amplitude (2mV) and energy concentration ( Figure 3B ) relative to the Control Group (1mV amplitude) ( Figure 3A ). Mismatches in muscle contraction force between the Control and the Caffeine with Treated Group are shown in figure 3 . Statistical differences (p<0.05) between the two portions analyzed can be seen, particularly in the graph depicting the strongest muscle contractions. Hence, caffeine affects skeletal muscle function, leading to an increase in mechanical performance by enhancing the ability of muscles to produce strength, work and energy.^( 27 )^ Therefore, improvements in motor skills can be attributed to caffeine, as advocated by other researchers.^( 28 )^

Caffeine increases myocardial activity, reducing the time of contraction and increasing the heart rate. It also has positive inotropic effects, given it increases contractile force.^( 29 )^ In this study, an increase in heart rate (bpm) ( Figure 4C ) and cardiac contraction force ( Figure 4D ) was observed in rats treated with caffeine. Combined, these effects translate into greater caloric expenditure, which indicates caffeine is in fact a thermogenic agent which enhances ergogenic effects.^( 30 )^ It has also been widely reported that moderate caffeine consumption (400mg to 600mg/day) is not associated with increased risk cardiovascular disease development. On the contrary, it seems to have a protective effect on the cardiovascular system. However, individuals predisposed to or suffering from cardiovascular diseases appear to be more sensitive to the effects of caffeine.^( 31 )^

## CONCLUSION

This study demonstrated the major electrophysiological changes observed in the central nervous system, myocardium and skeletal muscle after intraperitoneal injection of caffeine. Electrophysiological changes described in this study support acute positive effects on individual levels of attention, as observed following consumption of caffeinated drinks in order to maintain alertness. At the level of the central nervous system, there was an increase in delta, theta and alpha amplitude spectra, which are associated with memory encoding and enhanced learning. With regard to effects on skeletal muscles, increased contraction of the gastrocnemius muscle was demonstrated, a clear indication of how caffeine can be used to enhance performance in some physical activities. Electrocardiographic changes observed after caffeine administration are primarily related to increased heart rate and higher energy expenditure. Descriptions of quantitative changes in measurements in this electrophysiological spectrum are of interest to further studies aimed at determining the optimal daily caffeine dose and further describing positive, negative and toxic effects associated with the use of this stimulant.
