# Supplementary material for: Characterization of electrocorticographic, electromyographic and electrocardiographic recordings after the use of caffeine in Wistar rats
Source: Einstein (Sao Paulo). 2021 Nov 3;19:eAO6417. doi: 10.31744/einstein_journal/2021AO6417 (PMC8577317; doi:10.31744/einstein_journal/2021AO6417)
Supplement: Translation

# Translation: Caracterização dos registros eletrocorticográficos, eletromiográficos e eletrocardiográficos após uso de cafeína em ratos Wistar

**Authors:** Diego Arthur Castro Cabral, Fernanda Myllena Sousa Campos, Maria Clara Pinheiro da Silva, João Paulo do Vale Medeiros, Paula dos Santos Batista, Giovanna Coutinho Jardim, Jéssica Lígia Picanço Machado, Leonardo Giovanni Castro Cabral, Vanessa Joia de Mello, Moises Hamoy

**Affiliations:** 1 BelémPA Brasil Universidade Federal do Pará, Belém, PA, Brasil.

**Keywords:** Cafeína, Eletrocorticografia, Eletromiografia, Eletrocardiografia, Sistema nervoso central, Ratos Wistar

## Abstract

**Objetivo:**

Descrever os perfis eletrocorticográficos, eletromiográficos e eletrocardiográficos para relatar os efeitos eletrofisiológicos da cafeína em ratos Wistar.

**Métodos:**

Foram utilizados ratos Wistar, machos, adultos, pesando de 230g a 250g. Os animais foram divididos nos seguintes grupos: Grupo 1, Controle com solução fisiológica 0,9% por via intraperitoneal (n=27), e Grupo 2, Tratado com Cafeína (50mg/kg intraperitoneal; n=27). Foram realizadas avaliações por eletrocorticograma, eletromiograma e eletrocardiograma.

**Resultados:**

Houve variações nas oscilações cerebrais (delta, teta, alfa, beta e gama) na faixa de frequência de até 40Hz após a aplicação de cafeína em ratos. Observou-se que as potências nas faixas das oscilações delta e teta foram preponderantes. A força de contração nos músculos estriado esquelético e cardíaco aumentou. A avaliação do eletrocardiograma demonstrou que a duração dos intervalos RR, QRS e QT foram menores na presença da cafeína.

**Conclusão:**

No sistema nervoso central, houve aumento dos espectros de amplitude delta, teta e alfa, que auxiliam na codificação das memórias e estão relacionados à melhora do aprendizado. Em relação à musculatura esquelética, demonstrou-se aumento da contração do músculo gastrocnêmio, uma clara indicação de como a cafeína pode ser usada para aumentar o desempenho em algumas atividades físicas. As alterações eletrocardiográficas observadas após a administração de cafeína estiveram relacionadas principalmente ao aumento da frequência cardíaca e do consumo de energia.

## INTRODUÇÃO

A cafeína é um estimulante do sistema nervoso central (SNC) da classe das metilxantinas, e a droga psicoativa mais usada no mundo. O uso da cafeína é motivado pelo aumento da concentração, da cognição e do desempenho físico.^( 1 )^A droga também pode ser usada para tratamento da apneia idiopática da prematuridade^( 2 )^e da depressão respiratória aguda^( 3 )^e para controle da dor.^( 4 )^Dados epidemiológicos sugerem que o consumo habitual de café tem efeito protetor contra as doenças de Parkinson e Alzheimer, além de favorecer a perda de peso.^( 4 , 5 )^

A cafeína é um antagonista não seletivo dos receptores de adenosina, que estimula a atividade motora e tem efeitos excitatórios e de reforço.^( 6 )^A paraxantina é o principal metabólito da cafeína em seres humanos e promove liberação significativa de dopamina em áreas do estriado.^( 7 )^

O efeito estimulante da cafeína encontra–se amplamente descrito na literatura, sobretudo em estudos comportamentais e bioquímicos.^( 1 , ^6^ , 8 )^Entretanto, estudos quantitativos, descrevendo o impacto de alterações eletrofisiológicas nos diferentes sistemas após a administração do estimulante, são escassos.

## OBJETIVO

Descrever as alterações eletrofisiológicas induzidas pela cafeína em ratos Wistar machos, com base em registros eletrocorticográficos, eletromiográficos e eletrocardiográficos.

## MÉTODOS

### Animais

Os animais foram obtidos do Biotério Central da Universidade Federal do Pará (UFPA) e alojados individualmente no Biotério Experimental do Laboratório de Farmacologia e Toxicologia de Produtos Naturais, de abril de 2019 a novembro de 2020. Os animais tinham acesso livre à água e a alimento, foram mantidos em ambiente com temperatura controlada (25°C a 28°C) e submetidos a um ciclo claro–escuro de 12/12 horas. Todos os procedimentos experimentais foram conduzidos em conformidade com os princípios de cuidados de animais de laboratório e aprovados pelo Comitê de Ética em Experimentação Animal (CEUA nº 2675110219).

Foram usados 54 ratos Wistar adultos machos, pesando entre 230g e 250g. Os animais foram alocados para um dos seguintes grupos: Grupo 1 (n=27), Controle, tratado com volume equivalente de solução fisiológica (salina a 0,9%, injeção intraperitoneal – IP), e Grupo 2 (n=27), Tratado com Cafeína (50mg/kg IP), conforme descrito por Marriott, 1968.^( 9 )^Os ratos foram submetidos à eletrocorticografia (ECoG), eletromiografia (EMG) e eletrocardiografia (ECG). Eletrodos implantados cirurgicamente foram utilizados para registro de sinal nos procedimentos de ECoG, EMG e ECG. A coleta dos dados foi realizada em grupos separados, no 5º dia após a cirurgia. As mensurações foram realizadas no mesmo dia, empregando–se o seguinte delineamento em blocos: os dados eletrocorticográficos foram coletados primeiro, seguindo–se dos dados eletrocardiográficos e, por último, dos dados eletromiográficos. Os dados foram coletados sem uso de anestesia. Os animais foram acomodados em caixas acrílicas medindo 60x50x20cm (comprimento, largura e altura, respectivamente).

### Substâncias químicas

As seguintes substâncias químicas foram utilizadas: cloridrato de cetamina (Laboratório Köing, Santana de Parnaíba, SP, Brasil), cloridrato de xilazina (Laboratório Vallée, Montes Claros, MG, Brasil), lidocaína (Laboratório Hipolabor, Sabará, MG, Brasil) e cafeína em pó (Sigma), na forma de cristais diluídos em solução fisiológica 0,9%.

### Procedimento cirúrgico para implante de eletrodos

O procedimento cirúrgico foi realizado sob anestesia geral com 5mg/kg de xilazina e 50mg/kg de cetamina. O sítio operatório foi anestesiado com lidocaína. Uma vez anestesiados, os animais foram colocados em um dispositivo esterotáxico e devidamente posicionados. O local de implantação foi tricotomizado, e uma incisão foi realizada através da pele e dos tecidos subcutâneos e musculares, até chegar ao crânio. Duas craniotomias pequenas foram, então, criadas, −0,96 e 1mm lateralmente à fontanela anterior (bregma), a fim de se obter acesso a cada um dos hemisférios cerebrais contendo o córtex motor, onde os eletrodos foram implantados na superfície do cérebro.^( 10 )^Um parafuso foi introduzido no local da craniotomia para fixação dos eletrodos com resina acrílica odontológica.

### Eletrocorticograma

O preparo dos animais e a implantação dos eletrodos para aquisição do ECoG foram realizados de acordo com relatos prévios.^( 11 )^Os eletrodos foram posicionados 0,96mm e 1mm lateralmente à coordenada estereotáxica relativa ao bregma, em cada um dos hemisférios contendo o córtex motor.^( 10 )^Os eletrodos de registro e referência foram posicionados no hemisfério direito e esquerdo, respectivamente. No 5º dia após a cirurgia, os eletrodos foram conectados a um sistema de aquisição de dados composto por um amplificador de alta impedância (P511, Grass Technologies), monitorado com osciloscópio (Protek, 6510).^( 12 )^A administração intraperitoneal de cafeína foi realizada 15 minutos antes do registro. Os dados foram digitalizados de forma contínua a uma frequência de 1kHz, por meio de computador equipado com placa de aquisição de dados (National Instruments, Austin, TX, Estados Unidos). Os dados foram armazenados em um disco rígido e processados empregando–se *software* específico (LabVIEWexpress). Todo o experimento foi realizado em gaiola de Faraday.

### Eletromiograma

Após a aquisição dos dados de ECoG, eletrodos conjugados foram implantados 0,5cm acima da inserção do músculo gastrocnêmio, conforme descrito na literatura.^( 13 )^A administração intraperitoneal de cafeína foi realizada 15 minutos antes do registro eletromiográfico. Os animais foram mantidos em uma caixa acrílica durante o procedimento de registro (duração de 10 minutos), sendo os eletrodos conectados a um amplificador Grass P511 monitorado com osciloscópio.^( 13 )^

### Eletrocardiograma

Os eletrodos foram inseridos ao longo do vetor relativo à derivação D2. O eletrodo de referência foi colocado na região do quarto músculo intercostal, próximo à axila direita, e o eletrodo de registro no 11º espaço intercostal, 1,5cm à esquerda da linha sagital mediana.^( 14 )^Após a admi nistração intraperitoneal de 50mg/kg de cafeína e um período de latência de 15 minutos, os dados eletrocardiográficos foram registrados por 10 minutos em cada animal, analisando–se as seguintes variáveis: amplitude (mV), frequência cardíaca (bpm), intervalo RR, intervalo PQ, intervalo QT e duração do complexo QRS.

### Análise dos dados eletrofisiológicos

Os gráficos de amplitude mostram a diferença de potencial entre o eletrodo de referência e o eletrodo de registro a uma frequência de amostragem de mil amostras por segundo. Os espectrogramas foram calculados empregando–se uma janela de Hamming com 256 pontos (256/1000 segundos), e cada quadro foi gerado com sobreposição de 128 pontos por janela. Para cada quadro, a distribuição de energia espectral (DES) foi calculada empregando–se o método do periodograma médio de Welch. O histograma de frequência foi gerado a partir do primeiro cálculo de DES do sinal empregando–se a janela de Hamming com 256 pontos sem sobreposição com a DES resultando em histograma construído com caixas de 1Hz. Os sinais registrados até a faixa de 50Hz foram analisados. As bandas de frequência foram analisadas da seguinte maneira: delta (1Hz a 4Hz), teta (4Hz a 8Hz), alfa (8Hz a 12Hz), beta (12Hz a 28Hz) e gama (28Hz a 40Hz).^( 15 )^

### Análise estatística

A normalidade dos dados e a homogeneidade de variâncias foram verificadas por meio do teste de Kolmogorov-Smirnov e do teste de Levene, respectivamente. Os dados foram expressos como médias e desvios–padrão. Valores de F e p foram apresentados quando pertinentes. O nível de significância adotado foi de p<0,05. As comparações entre os grupos foram realizadas por meio da análise de variância (ANOVA) bidirecional seguida pelo teste de Tukey, para comparações múltiplas. O teste *t* de Student foi utilizado para comparação dos dados de ECoG, EMG e ECG (potência nas diferentes faixas de frequência) entre os Grupo Controle e Tratado com Cafeína. A ANOVA foi realizada apenas para análise da preponderância do espectro de frequência entre animais do mesmo grupo. As análises estatísticas para detecção e remoção de *outliers* foram realizadas empregando–se o software GraphPad Prism, versão 8 (Graph–Pad Software Inc., San Diego, CA, Estados Unidos).^( 12 )^

## RESULTADOS

### A cafeína alterou o espectro de potência das ondas cerebrais

Alterações importantes foram observadas no traçado do ECoG após a administração de cafeína em relação ao traçado inicial ( Tabela 1 ). O registro obtido no Grupo Controle pode ser visualizado na figura 1A , que mostra aumento da amplitude e da intensidade de potência no espectrograma em frequências inferiores a 10Hz. O traçado do ECoG, após a administração de cafeína, visualizado na figura 1B , mostra maior distribuição de potência acima de 10Hz.

**Figura 1:** Registro dos eletrocorticogramas do Grupo Controle e do Grupo Tratado com Cafeína. A) Eletrocorticograma do Grupo Controle, com ampliação do traçado que revela intensidade de potência nas frequências abaixo de 10Hz; B) Registro eletrocorticográfico obtido após a administração intraperitoneal de 50mg/kg de cafeína onde há um predomínio nas frequências acima de 10Hz; C) Distribuição de energia espectral após a administração intraperitoneal de cafeína (50mg/kg) ou solução fisiológica (controle) e as suas respectivas oscilações cerebrais. Os dados foram analisados por comparação de médias, empregando-se o teste *t* seguido do teste de Mann-Whitney, com nível de significância de p<0,001 (n=9)

**Tabela 1:** Valores numéricos obtidos nos registros eletrocorticográficos realizados durante os experimentos

Grupo	Delta (1Hz-4Hz)	Teta (4Hz-8Hz)	Alfa (8Hz-12Hz)	Beta (12Hz-28Hz)	Gama (28Hz-30Hz)
Controle, mV^2^/Hzx10^-3^	0,003883±0,0009877	0,01440±0,002569	0,01176±0,001643	0,007670±0,001497	0,003218±0,002155
Tratado com Cafeína, mV^2^/Hzx10^-3^	0,01227±0,001844 *	0,03031±0,006081 *	0,02189±0,003489 †	0,01979±0,003748 †	0,01173±0,001083 †

* Médias <0,001 e

† p<0,05 em relação ao Grupo Controle.

Todos os valores de ondas delta, teta, alfa, beta e gama obtidos nos Grupos Tratado com Cafeína foram apresentados.

A distribuição das ondas cerebrais pôde ser visualizada nos traçados registrados antes e depois da administração de cafeína ( Figura 1C ). Observou–se aumento da potência em ondas de baixa frequência, principalmente até 40Hz (p<0,001). De forma geral, a maior amplitude do espectro de potência do sinal ficou entre 1Hz e 8Hz, que corresponde a ondas delta e teta (p<0,001).

O espectro de potência do Grupo Controle revelou a seguinte relação: teta > alfa > beta > delta = gama (p<0,05), ilustrada na figura 2A . No Grupo Tratado com Cafeína, o perfil dominante de oscilação foi: teta > alfa = beta > delta = gama (p<0,05) ( Figura 2B ).

**Figura 2:** Amplitude média de potência das oscilações cerebrais delta, teta, alfa, beta e gama nos Grupos Controle e Tratado com Cafeína (50mg/kg). A) Potências das ondas cerebrais predominantes no Grupo Controle; B) Potências predominantes nas oscilações cerebrais nos animais que receberam cafeína por via intraperitoneal; C) Comparação das oscilações detectadas em cada grupo (n=9)

A oscilação média da onda delta antes e após a administração de cafeína (0,003883±0,0009877mV^2^/Hzx10^–3^e 0,01227±0,001844mV^2^/Hzx10^–3^, respectivamente) diferiu de forma significante (p<0,0001), sendo maior no Grupo Tratado com Cafeína. No Grupo Controle, as oscilações médias nas faixas teta (0,01440±0,002569mV^2^/Hzx10^–3^) e alfa (0,01176±0,001643mV^2^/Hzx10^–3^) diferiram de forma significante em relação ao Grupo Tratado com Cafeína (teta, 0,03031±0,006081mV^2^/Hzx10^–3^e alfa 0,02189±0,003489 mV^2^/Hzx10^–3^; p<0,001). A oscilação média na faixa beta no Grupo Controle (0,007670±0,001497mV^2^/Hzx10^–3^) diferiu de forma significante em relação ao Grupo Tratado com Cafeína (0,01979±0,003748mV^2^/Hzx10^–3^; p<0,001). Na faixa de oscilação gama, a média foi de 0,003218±0,002155mV^2^/Hzx10^–3^no Grupo Controle e de 0,01173±0,001083mV^2^/Hzx10^–3^no Grupo Tratado com Cafeína (p<0,001).

### A cafeína aumentou a amplitude de contração da musculatura esquelética estriada

O registro eletromiográfico apresentado na figura 3A e na tabela 2 mostra o padrão de contração muscular em baixa amplitude (até 1mV), com espectrograma de distribuição de energia até 50Hz. Passados 15 minutos da administração intraperitoneal de cafeína, a frequência de contrações aumentou, com registro de amplitude acima de 2mV e concentração mais alta de energia, como mostra a figura 3B .

**Figura 3:** Registro eletroneuromiográgico da contração do músculo gastrocnêmio. O tempo de registro foi de 300 segundos. A) Registro dos animais do Grupo Controle; B) Registro após a administração de cafeína; C) A potência observada no registro completo em frequências de até 50Hz demonstra a potência das contrações musculares mensuradas nos animais controle e tratados com cafeína; D) Potência das contrações mais fortes registradas nos Grupos Controle e Tratado com Cafeína, em tempo fixo de 5 segundos de contração (n=9)

**Tabela 2:** Representação numérica dos valores obtidos durante o estudo eletromiográfico

Grupo	Controle (mV^2^/Hzx10^-3^)	Cafeína (mV^2^/Hzx10^-3^)
Registro EMG completo	6,676±2,702	28,22±6,736
Registro da contração mais forte (EMG)	12,94±4,470*	78,56±26,46 †

** Médias p<0,05 e

† <0,01 em relação ao Grupo Controle.

EMG: eletromiografia.

Todos os valores obtidos nos Grupos Controle e Tratado com Cafeína foram apresentados. Valores encontrados no estudo de eletromiografia e valores referentes exclusivamente à contração mais forte do músculo gastrocnêmio também foram apresentados.

As diferenças de amplitude entre o total de registros até 50Hz mostradas na figura 3 indicam que a potência média diferiu de forma significante entre o Grupo Controle e o Tratado com Cafeína (6,676±2,702mV^2^/Hzx10^–3^e 28,22±6,736mV^2^/Hzx10^–3^, respectivamente; p<0,001) ( Figura 3C ). A análise das contrações mais fortes registradas no Grupo Controle e no Grupo Tratado com Cafeína revelou diferença significante (12,94±4,470mV^2^/Hzx10^–3^e 78,56±26,46mV^2^/Hzx10^–3^, respectivamente; p<0,001) ( Figura 3D ).

### A cafeína alterou os parâmetros eletrocardiográficos

As principais alterações eletrocardiográficas observadas após a administração de cafeína foram relacionadas ao aumento da frequência cardíaca, como mostrado nas figuras 4A, B, C e D . Houve aumento significante da frequência cardíaca após o uso da cafeína (249,2±21,26bpm e 303,7±7,194bpm, para os Grupos Controle e Tratado com Cafeína, respectivamente; p=0,0004) ( Figura 4E e Tabela 3 ).

**Figura 4:** Eletrocardiograma dos animais e ampliação do traçado eletrocardiográfico. A) Traçado do Grupo Controle na derivação D-II; B) Eletrocardiograma do Grupo Tratado com Cafeína na derivação D-II; C) Ampliação do intervalo de 40 a 50 segundos do Grupo Controle; D) O intervalo entre 40 e 50 segundos do eletrocardiograma dos animais tratados com cafeína está ampliado; E) Traçado do eletrocardiograma dos ratos em ritmo sinusal na derivação D-II, mostrando os intervalos analisados, 1 segundo de aumento; F) Traçado eletrocardiográfico do Grupo Tratado com Cafeína, demonstrando as características da deflagração cardíaca e encurtamento do intervalo R-R, 1 segundo de aumento

**Tabela 3:** Apresentação numérica dos valores obtidos durante a execução do estudo eletrocardiográfico

Parâmetro	Frequência cardíaca (bpm)	Amplitude (mV)	Intervalos RR (segundos)	Duração QRS (segundos)	Intervalos QT (segundos)	Intervalos PQ (segundos)
Controle	249,2±21,26	0,4746±0,04072	0,3453±0,01276	0,01011±0,001364	0,0570±0,004062	0,07056±0,003779
Tratado com Cafeína	303,7±7,194 *	0,5250±0,01949 †	0,2400±0,03651 *	0,0082±0,0007953 †	0,03153±0,004996 *	0,08306±0,007435 *

* p<0,001 em relação ao Grupo Controle e

† p<0,01 em relação ao Grupo Controle.

Lista de parâmetros obtidos dos registros eletrocardiográficos dos Grupos Controle e Tratado com Cafeína (teste *t* de Student e teste de Mann-Whitney).

A amplitude média também aumentou de forma significante após a administração de cafeína (0,4746±0,04072mV e 0,5250±0,01949mV para Grupo Controle e Grupo Tratado com Cafeína, respectivamente; p=0,0061). O aumento da frequência cardíaca no registro revelou encurtamento do intervalo RR no ECG ( Figuras 4E e F ); as médias diferiram de forma significante entre o Grupo Controle e o Grupo Tratado com Cafeína (0,3453±0,01276 segundos e 0,2400±0,03651 segundos, respectivamente; p=0,0004). A duração média do complexo QRS também foi caracterizada pela diminuição do tempo de execução e diferiu de forma significante entre o Grupo Controle e o Grupo Tratado com Cafeína (0,01011±0,001364 segundos e 0,0082±0,0007953 segundos, respectivamente; p=0,0062). O ciclo cardíaco representado pelo intervalo QT, que envolveu o período de despolarização e repolarização ventricular, também diferiu estatisticamente (média de 0,0570±0,004062 segundos e 0,03153±0,004996 segundos no Grupo Controle e no Grupo Tratado com Cafeína, respectivamente; p=0,0004). No caso do intervalo PQ, a média foi de 0,07056±0,003779 segundos no Grupo Controle e de 0,08306±0,007435 segundos no Grupo Tratado com Cafeína ( Tabela 3 ).

## DISCUSSÃO

Neste estudo, os registros de ECoG, EMG e ECG foram empregados para descrever alterações eletrofisiológicas observadas em ratos após a administração de cafeína. No Grupo Tratado com Cafeína, a potência média foi 50% mais alta na amplitude das oscilações cerebrais delta em relação ao controle. Acredita–se que as ondas delta contribuam para a codificação de memórias e para o aprendizado.^( 16 )^Efeitos agudos positivos sobre a atenção também foram demonstrados na maioria dos estudos que investigaram os efeitos da cafeína sobre a cognição.^( 17 )^Esse achado também pode estar relacionado ao estágio delta, uma vez que a atividade delta modula o desempenho mental por meio da inibição de estímulos não relacionados à tarefa a ser executada, aumentando o nível individual de atenção durante a execução de tarefas que requerem processamento cerebral interno cuidadoso.^( 18 )^

O ritmo teta é implicado em diversas atividades, como estabelecimento de padrões de palavras para reconhecimento da fala e sincronização de movimentos de microssacadas, frequentemente observados no contexto exploratório e atento e na aprendizagem implícita, uma categoria de aprendizagem em grande parte inconsciente e independente do hipocampo.^( 19 – 21 )^

As ondas alfa guardam relação com o processamento cognitivo e a autorregulação e aumentam em situações associadas a ganhos de atenção.^( 22 , 23 )^As oscilações beta são um forte preditor do desempenho perceptivo e motor.^( 24 )^Essas oscilações têm relação com estados de alerta, foco e pensamento ativo.^( 25 )^No córtex superior, as ondas gama aumentam durante o uso da memória de trabalho e aprendizagem. Essas oscilações participam da comunicação neural, refletindo a transferência de informações do mundo exterior para o encéfalo.^( 26 )^

O aumento da amplitude de todas as ondas cerebrais neste estudo ( Figura 1C ) sugere que a cafeína ou qualquer de seus metabólitos pode afetar direta ou indiretamente as vias envolvidas na geração desses ritmos, podendo estimular as funções cognitivas associadas às oscilações cerebrais e corroborando o fato de que a cafeína age como um estimulante do SNC.^( 6 )^

Neste estudo, a administração intraperitoneal de cafeína aumentou a frequência de contração do músculo gastrocnêmio, registrando–se maiores amplitude (2mV) e concentração de energia ( Figura 3B ) em relação ao Grupo Controle (amplitude de 1mV) ( Figura 3A ). As disparidades de força de contração muscular entre os Grupos Controle e Tratado com Cafeína encontram–se ilustradas na figura 3 . Diferenças significativas (p<0,05) entre as duas porções analisadas podem ser observadas, sobretudo no gráfico que mostra as contrações musculares mais potentes. Assim, a cafeína pode afetar a função da musculatura esquelética, promovendo maior desempenho mecânico por meio do aumento da capacidade do músculo de produzir força, trabalho e energia.^( 27 )^Portanto, a melhora das habilidades motoras pode ser atribuída à cafeína, conforme descrito por outros pesquisadores.^( 28 )^

A cafeína estimula a atividade miocárdica, o que diminui o tempo de contração e resulta no aumento da frequência cardíaca. Além disso, ela possui efeito inotrópico positivo, uma vez que aumenta a força de contração.^( 29 )^Neste estudo, a frequência cardíaca e a força de contração cardíaca ( Figura 4C ) aumentaram nos ratos tratados com cafeína. Combinados, esses efeitos se traduzem em maior gasto calórico, o que confere à cafeína o *status* de agente termogênico, capaz de intensificar os efeitos ergogênicos.^( 30 )^Além disso, o conceito de que o consumo moderado de cafeína (400mg a 600mg ao dia) não aumenta o risco de desenvolvimento de doença cardiovascular é amplamente difundido; ao contrário, parece haver um efeito protetor sobre o sistema cardiovascular. Entretanto, indivíduos predispostos ou que sofrem de doenças cardiovasculares parecem ser mais sensíveis aos efeitos da cafeína.^( 31 )^

## CONCLUSÃO

Este trabalho demonstrou as principais alterações eletrofisiológicas observadas no sistema nervoso central, no miocárdio e na musculatura esquelética após a injeção intraperitoneal de cafeína. As alterações eletrofisiológicas aqui descritas corroboram os efeitos agudos positivos sobre o nível individual de atenção, conforme observado após o consumo de bebidas cafeinadas com intuito de manter o estado de alerta. No sistema nervoso central, observou–se aumento dos espectros de amplitude delta, teta e alfa, associados à codificação de memórias e à melhora da aprendizagem. No que se refere à musculatura esquelética, foi demonstrado aumento da contração do músculo gastrocnêmio, uma indicação clara de como a cafeína pode ser utilizada para melhorar o desempenho em algumas atividades físicas. As alterações eletrocardiográficas observadas após a administração de cafeína se referem principalmente ao aumento da frequência cardíaca e do consumo de energia. A descrição de alterações quantitativas de mensurações realizadas nesse espectro eletrofisiológico é de interesse para estudos futuros destinados a determinar a dose diária ideal de cafeína e a descrever com mais detalhes os efeitos positivos, negativos e tóxicos associados ao uso desse estimulante.
